# *Stachytarpheta jamaicensis* (L.) Vahl: From Traditional Usage to Pharmacological Evidence

**DOI:** 10.1155/2016/7842340

**Published:** 2016-01-26

**Authors:** Pearl Majorie Liew, Yoke Keong Yong

**Affiliations:** Department of Human Anatomy, Faculty of Medicine and Health Sciences, Universiti Putra Malaysia (UPM), 43400 Serdang, Selangor, Malaysia

## Abstract

*Introduction*.* Stachytarpheta jamaicensis* (L.) Vahl belongs to the family of Verbenaceae and is commonly known as Gervao, Brazilian tea, verbena cimarrona, rooter comb, or blue porter weed. It is one of the important plants with high medicinal and nutraceutical benefits.* S. jamaicensis* contains various medicinal properties in traditional and folk medicinal systems, with cures for several diseases.* Objective*. The objective of this review paper is to collect information concerning the morphology, distribution, traditional usage, phytochemical compositions, biological activities, and safety data of* S. jamaicensis*.* Materials and Methods*. The information was obtained from literature search through electronic databases such as PubMed and Google Scholar on* S. jamaicensis*.* Results and Conclusion*. The high medicinal properties of this plant, for instance, antimicrobial and antifungal effect as the main activities, but verbascoside as the main active chemical component, make it a valuable source of the medicinal compound. This review paper summarizes all information concerning the morphology, distribution, traditional usage, phytochemical compositions, pharmacological activities, and toxicological studies of* S. jamaicensis*.

## 1. Introduction

For decades, medicinal plants have been utilized as a natural source containing bioactive compounds that offer therapeutic benefits and affordable treatments against a wide spectrum of diseases. The usage of medicinal plants as an alternative to chemically synthesized drugs in the treatment of diseases has been accepted on a global scale. Due to the use and efficacy of medicinal plants, studies have extensively demonstrated that these medicinal plants contain secondary metabolites and have reported their role in various medicinal properties, including analgesic, antidiarrheal, antimicrobial, antioxidant, antihypertensive, antinociceptive, and anti-inflammatory [1–6].

*Stachytarpheta jamaicensis *(L.) Vahl is a member of the family of Verbenaceae and is commonly known as Gervao, Brazilian tea, verbena cimarrona, rooter comb, or blue porter weed [1, 7]. This plant mostly grows in the tropical regions of America, as well as in the subtropical forests of Africa, Asia, and Oceania.* S. jamaicensis *has been an important medicinal plant with great medicinal properties in traditional and folk medicinal systems. This plant has been used traditionally by the elderly as a cure for allergies and respiratory conditions, cough, cold, fever, constipation, digestive complications, and dysentery and promotes menstruation, among others [8].

Herein, this present review paper aims to document the morphology, distribution, traditional usage, phytochemical compositions, and medicinal properties of* S. jamaicensis*, as well as the future prospects for further investigation in developing effective traditional-ethno drugs in the pharmaceutical industry.

## 2. *Stachytarpheta jamaicensis* (L.) Vahl

*S. jamaicensis *have been used traditionally by the elderly for decades. Nevertheless, little is known about this plant compared to other plants.* S. jamaicensis *is a weedy herbaceous plant that grows 60–120 cm tall. This plant has a smooth, dark green coloured stem, which turns woody towards the base of the stem [9].* S. jamaicensis *normally reproduces flowers in mix of bluish and pinkish colours or could bear flowers with a purple to deep blue colour. The leaves are opposite, greyish green in colour, have a smooth surface, and have a round apex and distinct petioles [10, 11]. The plant mostly grows in the tropical regions of America and other subtropical forests such as in Nigeria, Europe, and Russia [2]. It is also widely distributed in acclimatized tropics such as those in Malaysia and Indonesia. It is referred to as “Jolok Cacing” or “Selasih Dandi” in these countries [12].

## 3. Traditional Usage of* Stachytarpheta jamaicensis*

*S. jamaicensis *is widely known for its high medicinal importance in traditional and folk medicinal systems in various countries. This plant has been reported to possess pharmacological effects due to the presence of various bioactive phytochemicals.

In herbal medicine,* S. jamaicensis *itself has been known to demonstrate antacid, analgesic [13], anti-inflammatory [12], hypotensive [3], antihelminthic [14], diuretic, laxative, lactagogue, purgative, sedative, spasmogenic, vasodilator, vulnerary, and vermifuge properties [1, 8, 15].

*S. jamaicensis *has also been extensively used by the elderly as a cooling tonic for the stomach. The leaf and stem extracts of this plant are usually prepared in the form of tea bag before being consumed. This cooling tonic is consumed to stimulate the function of the gastrointestinal tract or to aid in digestive problems such as indigestion, acid reflux, ulcers, constipation, dyspepsia, and slow digestion [1]. Furthermore, it is also often used to treat allergies and respiratory conditions such as asthma, cold, the flu, bronchitis, and cough, as well as cirrhosis and hepatitis [7]. The leaf extract of* S. jamaicensis *can also be applied externally to clean cuts, wounds, ulcers, and sores [7].

In Southern Nigeria,* S. jamaicensis *is practiced by women to treat menstrual disorders and female complaints [7]. The leaves are boiled in water and served as a tea for the women to drink after childbirth in order to restore the uterus back to its original position in the body. It is also known to regulate hormone and increase milk supply in breast-feeding mothers [7]. The leaves of this plant are also taken orally to treat dysentery and intestinal worms as a juice [7]. However, it is not recommended for pregnant women or individuals with low blood pressure to consume* S. jamaicensis*, since it is considered to have abortive and hypotensive effect [7].

## 4. Phytochemical Constituents of* Stachytarpheta jamaicensis*

*S. jamaicensis *is rich in secondary metabolites, commonly known as bioactive compounds. Nowadays, these bioactive compounds are discovered to be responsible in exhibiting their therapeutic activities. There are several major groups of secondary metabolites that are present in the plant, including alkaloids, flavonoids, phenols, steroids, and terpenoids. These bioactive compounds can be found abundantly in all parts of the plant. In particular, the phytochemicals in phenolic compounds of* S. jamaicensis*, which include coumarins, flavonoids, tannins [15], and saponins [1], are the most studied among researchers due to their therapeutic properties. Eventually, these compounds produce different medicinal properties. For example, the phytochemicals present in* S. jamaicensis *(saponins, tannins, and flavonoids) have been known to exhibit antimicrobial activity on* E. coli*,* C. albicans*,* S. aureus*, and* P. vulgaris* [1]. The presence of phytochemical constituents in different parts of* S. jamaicensis *is summarized in Table 1, according to several research works. Negative results stated in the table do not indicate that the compound is absent but are rather due to low concentrations of this compound. Apart from that, few pure compounds have been isolated and determined in* S. jamaicensis* plant (Table 2).

## 5. Pharmacological Activities

### 5.1. Antimicrobial and Antifungal Activities

New infectious diseases emerge continuously and account for a high proportion of health complications that affect the human population throughout developing countries. It has been reported that the main reason that explains this worsening situation is because of antibiotic-resistance microorganisms. According to Putera and Anis Shazura [15], microorganisms have gained and developed their resistance against antibiotics through genetic alterations between themselves and other organisms. Consequently, immense therapy problems in the treatment of infectious diseases have arisen, and alternative tactics are needed to fight against this world concerning threat. This has led researchers to search for substitutes from different sources, including medicinal plants.

*S. jamaicensis *has been used traditionally by the elderly for the treatment of infectious diseases [15]. Besides that, it has been reported extensively that this plant is capable of sustaining the microbial activity of a broad range of pathogenic microorganisms [1, 16]. Numerous studies have reported and proven the antimicrobial potential of* S. jamaicensis *extracts towards pathogenic microorganisms, including bacteria and fungi [1, 16–19].

A study conducted by Putera and Anis Shazura [15] has investigated the antimicrobial activity of the* S. jamaicensis *plant (including roots, stem, and leaves) by using the disk diffusion technique. The crude plant extracts in all three parts of the plant have exhibited antimicrobial activity against* Pseudomonas aeruginosa, Micrococcus luteus*, and* Escherichia coli*. The crude root extract showed better antimicrobial activity against* Pseudomonas aeruginosa *compared to other crude extracts, but in a dose-dependent manner [15].

The antimicrobial activity of* S. jamaicensis *leaf extract was also reported using gutter and punch hole methods [1]. The results suggest that the aqueous extract of* S. jamaicensis *(L.) Vahl inhibits the growth of* Bacillus subtilis*,* Escherichia coli*,* Candida albicans*,* Staphylococcus aureus*,* Pseudomonas aeruginosa*,* Proteus vulgaris*,* Klebsiella aerogenes*, and* Proteus mirabilis *[1]. The ethanol extract of* S. jamaicensis *(L.) Vahl showed activity on* Bacillus subtilis*,* Escherichia coli*,* Candida albicans*,* Pseudomonas aeruginosa*, and* Proteus mirabilis* [1]. Both these two extracts were found to possess their activity at high concentrations, which suggests that it is a possible antibiotic that should be administered at high doses.

The antifungal activity of* S. jamaicensis *was evaluated against isolated fungi using the agar well diffusion bioassay. Thomas et al. [16] revealed that the petroleum ether extract of* S. jamaicensis *exhibited its fungicidal activity against two of the tested fungi, namely,* Curvularia *sp. and* Penicillium *sp. On the other hand, the methanolic extract showed effectiveness against* Curvularia *sp.,* Penicillium *sp., and* Fusarium *sp. Overall,* S. jamaicensis *does possess antifungal effects [16].

Suneetha et al. [17] assessed the antimicrobial and antifungal efficacy of phenolic leaf extracts of different medicinal plants, including* S. jamaicensis*, in their comparative study. The study was tested against gram positive and gram negative strains, as well as three different fungal strains. The phenolic extract of* S. jamaicensis *exhibited maximum to moderate antimicrobial activity against* Pseudomonas florescence*,* Staphylococcus aureus*, and* Bacillus subtilis*, and the least in* Escherichia coli*. Individual plants exhibited various fungicidal activities against different fungi, and thus,* S. jamaicensis *was found to be more susceptible to* Mucor *sp. compared to the other two fungi [17]. The antifungal efficacy of this plant is believed to be attributable to the presence of phenolic compounds and to administration in high concentration [17].

The antibacterial property of the leaf extract of* S. jamaicensis *was detected against the serotyped strain,* Staphylococcus aureus*, using a disk diffusion method [18]. The result indicated the zone of inhibition against* S. aureus *increased gradually in a dose-dependent manner, and the highest zone of inhibition was observed in the highest concentration (10 mg/mL) [18].

### 5.2. Antioxidant Properties

Due to the presence of an unpaired electron, most of the free radicals are very unstable and highly reactive. Free radicals or oxidants are derived from one's bodily metabolic processes, or from the exposure to external sources such as X-rays, cigarette smoking, or industrial chemicals [20]. Free radicals, for instance, hydrogen peroxide, nitric oxide radical, superoxide anion radical, and peroxynitrite radical, are capable of attacking and damaging proteins, carbohydrates, lipids, and even nucleic acids [20]. Consequently, a balance between free radicals and antioxidant is crucial for proper physiological functionality. Otherwise, it could lead to a number of human diseases.

Vegetables, fruits, medicinal plants, and herbal infusions have been known as important sources of antioxidants [21]. Numerous medicinal plants contain large amounts of antioxidant compounds such as phenolic compounds and polyphenols, which play an important role in eliminating free radicals [21]. Due to antioxidants, it is important for the prevention and treatment of free radical related disorders. Interest has increased considerably in finding naturally occurring antioxidants for use in foods or medicinal materials.

Álvarez et al. [22] documented that ethyl acetate extract of* S. jamaicensis*' leaves significantly suppresses reactive oxygen species (ROS) production by inhibiting XO and scavenge ROS. In contrary, hexane extract does not show any antioxidant activity. The antioxidant activity in acetate extract may attribute by the presence of catechins (a type of flavonoid) in the leaves. Apart from that, Sivaranjani et al. [23] conducted an extensive antioxidant assay on various extracts of* S. jamaicensis* leaves* in vitro*. Among all the extracts, methanolic extract was reported to be more effective in total antioxidant activity, DPPH and FRAP. This may due to the high concentration of phenol and flavonoids presented in the methanolic extract. As documented, compounds with antioxidant properties are mainly from phenolic acids, flavonoids, and polyphenols [24]. In addition, ethanolic leaf extract of* S. jamaicensis* also proved to improve the catalase activity on alloxan-induced diabetic rats [25]. All of the results obtained from above suggested that* S. jamaicensis* could be a potential pharmaceutical value in the treatment of oxidative stress related health diseases.

### 5.3. Anti-Inflammatory and Antinociceptive Activities

Inflammation can be characterized as a pathophysiological reaction to injury or infection that leads to redness, warmth, swelling, pain, and loss of function. Inflammation is an important body defense system that eliminates foreign bodies or pathogens and prevents further damage to the host. However, if the harmful inflammation goes untreated, it will lead to the root of many diseases such as sepsis, atherosclerosis, and cancer formation. Many nonsteroidal anti-inflammatory drugs (NSAIDs) are readily available nowadays, and they are the medications that have been widely used to treat a wide range of inflammation problems, from just a light headache, to rheumatoid arthritis. The frequent usage of NSAIDs causes unpleasant side effects, including indigestion, stomach ulcers, and allergic reactions [26]. Apart from that, the long term consumption of NSAIDs is reported to be associated with heart attack, stroke, or heart failure [27]. Numerous herbal medicines may exhibit anti-inflammatory effects or pain relief activities. Moreover, its side effects are much less than the NSAIDs. Due to this, ethnomedicine has become an interest to scientists around the world in the search for new anti-inflammatory and antinociceptive drugs.

Based on the study by Sulaiman and his group [12], ethanolic extract of* S. jamaicensis* leaves exhibits potential anti-inflammatory and analgesic activities in the animal setting. The results suggested that* S. jamaicensis* leaf extract possesses antinociceptive activity against chemically and thermally induced nociception and against both inflammation- and non-inflammation-mediated nociception via suppression of both peripheral and central levels. In addition, they also proved that* S. jamaicensis* leaf extract exhibited anti-inflammatory activity in both acute and chronic models of inflammation. This activity may be due to inhibition of arachidonate COX, which is consistent with antinociceptive studies [12]. Similar studies by Jagadish and Gopalkrishna [13] also reported that the ethanolic extract of* S. jamaicensis* exhibited analgesic activity in an acetic acid-induced writhing test and tail immersion method on Swiss albino mice.

### 5.4. Antidiarrheal Activity

Diarrhea is one of the most common factors that have caused several millions of deaths worldwide. Children under the age of five and infants younger than one year account for more than half of these deaths [28]. Diarrhea is known as the condition with loose bowel movements and often characterized by watery and increased frequency of stools [29]. Antidiarrheal activity is one of the medicinal properties exhibited by* S. jamaicensis*. This plant has been used as an agent in folk medicine in the management of diarrhea.

Evaluation of antidiarrheal activity by* S. jamaicensis *was investigated by Álvarez et al. [22]. This research was performed using the methanol extract of the leaves of* S. jamaicensis *in castor oil and magnesium sulphate-induced (causative organisms of diarrhea) diarrhea models* in vivo*. The evidence from this study has shown that the methanol extract of* S. jamaicensis *possessed a significantly high volume of antidiarrheal activity in Swiss albino mice, in a dose-dependent manner [30].

### 5.5. Antihypertensive Activity

Hypertension has contributed as one of the most common diseases, with high rates of morbidity and mortality all over the globe.* S. jamaicensis *is claimed to have an antihypertensive effect, but there is a lack of information available on this [31]. Thus, an* in vivo *study has been conducted to verify the antihypertensive property of* S. jamaicensis*.

According to the research done by Idu et al. [32], it was concluded that the aqueous extract of* S. jamaicensis *demonstrated a significant dose-dependent antihypertensive effect. The extract reduced blood pressure and heart rates of anaesthetised rabbits, gradually in increasing doses, and the maximum effect was with the highest doses [32].

### 5.6. Antidyslipidaemia Activity

Cardiovascular diseases are among the major world concerning threats that afflict humans and are responsible for millions of deaths. The factors for the development of cardiovascular disease can vary. One factor is dyslipidaemia. This may be either primary or associated with other complications such as hypertension, diabetes mellitus, and obesity, which contribute to abnormalities such as elevated plasma levels of triglycerides, LDL and VLDL cholesterol, and low HDL cholesterol levels [33–35].

In view of this aspect, an* in vivo *study has been undertaken to evaluate the effect of* S. jamaicensis *on lipid-lowering property. In this study, it was found that aqueous infusion (tea) of* S. jamaicensis *significantly decreased the plasma levels of triglycerides, total cholesterol, LDL, and VLDL but increased HDL cholesterol levels in the treated animal models [36].

### 5.7. Hepatoprotective Effect

The hepatoprotective effect of* S. jamaicensis *was assessed by carbon tetrachloride (CCl_4_) induced toxicity with the hepatoprotective drug (Liv-52) in albino rats [37]. The blood of the rats was collected from the carotid artery. Serum was separated and screened for the estimation of various biochemical parameters (serum glutamic oxaloacetic transaminase (SGOT) also known as ASAT, serum glutamic-pyruvic transaminase (SGPT) also known as ALAT, serum alkaline phosphatase (SALP), and serum bilirubin and reversed total protein). The results showed that there was a reduction in CCl_4_-induced elevated levels of SGPT, SGOT, SALP, and serum bilirubin and reversed total protein in the ethanolic extract of* S. jamaicensis *treated animals [3]. These findings indicate that the leaves of* S. jamaicensis *show potent hepatoprotective benefits.

### 5.8. Wound Healing

The wound healing effect of the hydroalcoholic leaf extract of* S. jamaicensis *was evaluated in the study done by Pandian et al. [37]. The wound healing activity was carried out on streptozotocin-induced diabetic albino Wistar rats by using two methods: excision and dead space models. The results indicated that the group of rats treated with hydroalcoholic leaf extract significantly increased in terms of wound healing parameters (wound closure percentage, tensile strength, hydroxyproline, hexosamine, DNA, and total protein content), along with a significant reduction in the period of epithelialization and blood glucose levels, as compared to untreated animals in a dose-dependent manner [38]. In a similar study, Rozianoor and her team also proved that* S. jamaicensis* ethanolic leaf extract is capable of enhancing wound healing on alloxan-induced diabetic rats [25]. It was suggested that hydroalcoholic leaf extract of* S. jamaicensis *may have potential wound healing and hypoglycaemic properties [37].

### 5.9. Other Pharmacological Properties

Apart from the abovementioned pharmacological activities,* S. jamaicensis* has been documented to exhibit insecticidal [39], larvicidal [38], and antihelmintics activities [40]. In addition,* S. jamaicensis* was also demonstrated to have antityphoid activity [41] and anti-HIV1 reverse transcriptase inhibitory activity [42], as well as to treat cancer-like symptoms [43].

## 6. Toxicology Studies of* S. jamaicensis*

Although it was known that phytotoxicity is very low, the toxicological assessment must be performed on all the herbal medicine. Not only does the data obtained from toxicological studies help in determining the upper limits of administration, it is also mandatory for the registration of the product with National Health Authorities. Toxic effects can range from negligible to so severe as to preclude further development of the compound [44]. There are few toxicity studies being conducted on* S. jamaicensis*. Study from Idu et al. [45] documented that rats were administered with 25, 50, and 75 g of powdered* S. jamaicensis* leaves showing no significant alteration in the normal serum biochemistry when compared with control. Moreover, ultrasound picture of heart, liver, kidney, and spleen also showed no significant difference from control [45]. However, Ataman et al. [46] reported that rats were fed with the same dose and concentration as in Idu et al. [45] showed slight variation on the physical signs/body appearance of the animals and mild histopathologic lesions such as congestion, fatty changes, and necrosis were found in certain tissues, such as liver, blood vessels, kidney, lung, and testis. On the other hand, the brain, eyes, small and large intestines, and heart tissues were essentially normal. These data suggested that* S. jamaicensis* seems to cause mild systemic toxicity in certain tissues [46]. Further studies have been conducted by Idu et al. [1] on acute toxicity of* S. jamaicensis* leaves. The results revealed that aqueous extract of* S. jamaicensis* showed no toxic toward Wistar rats even up to dose of 4 g/kg body weight and no significant changes in body weight on Wistar rats. In addition, the color of the eyes was normal and loss of hair was absent in the animal [1]. Recent study reported that the body weight and liver of albino rats fed with 25, 50, and 75 g of powdered* S. jamaicensis* leaves showed no significant difference to that of the control group [47]. Overall data showed that high dose of the* S. jamaicensis* leaves extract does not show toxicity and it is relatively safe for therapeutic cure of diseases. However, further investigation is needed to confirm and prove on its chronic toxicity effect.

## 7. Future Prospect and Conclusion

It is quite evident that* S. jamaicensis *is an important medicinal plant that plays a vital role in medicinal systems, particularly in traditional and folk medicinal systems.* S. jamaicensis *has also been proven to have a number of phytochemical constituents, which are the key factors to exhibit high medicinal values of this plant. Mostly, all parts of this plant (leaves, stem, and root) are used as agents to cure a variety of diseases. As the pharmaceutical industry is looking forward to developing new drugs from natural sources, the development of effective traditional-ethno drugs from* S. jamaicensis *can be considered in the treatment of diseases by exploiting the main pharmacological activities. A systemic research and development work should be undertaken for the development and introduction of new products into the community, with the aim for a better economic and therapeutic utilization.

## Figures and Tables

**Table 1:** A summary of phytochemical constituents present in different parts of *S. jamaicensis* (L.) Vahl (aerial, leaf, stem, and root) [1–3, 15, 17, 37, 48, 49].

Phytochemical compositions	Plant part			
	Leaf extract	Stem extract	Root extract	Aerial extracts
Alkaloids	+	+		+
Amino acids	−	−		−
Anthraquinone	−	−		
Carboxylic acids				−
Carbohydrates	+	+		+
Coumarin	−	−	−	−
Flavonoids	+	+	+	+
Glycoside	+			+
Phenol	+	+	+	−
Phlobotanin	+	−	−	
Proteins	+	+		
Quinone	+			−
Saponins	+	−	+	+
Starch	−	−		
Steroids	+			
Tannins	+	+	+	+
Terpenoids	+	+	+	−

+ = present; − = absent.

**Table 2:** A summary of pure compounds being isolated from *S. jamaicensis* (L.) Vahl.

Bioactive compounds	References	
Tarphetalin	[50]	
Choline		
Phenolic acids		
Chlorogenic acid		
Catechuic tannins	[51]	
6-Hydroxyluteolol 7-glucuronide		
Luteolol 7-glucuronide		
Apigenol 7-glucuronide		
n-C_29_H_60_		
n-C_30_H_62_		
n-C_32_H_66_		
n-C_31_H_64_		
n-C_33_H_68_		
n-C_34_H_70_	[52]	
n-C_35_H_72_		
*α*-Spinasterol		
Saturated aliphatic ketone		
Saturated aliphatic carboxylic acid		
Unsaturated hydroxy carboxylic acid		
Iridoid ipolamiide	[53]	
Phenylpropanoid glycoside—verbascoside		
Lanostane phenylacetate (1,3,16-*β*-yl-phenypropylacetate-lanostan-5,11,14,16,23,25-hexen-22-one)	[54]	
16-*β*-(*β*-D-Glucopyranosyl-3,8,22-trihydroxy)-cholestan-1-*β*-yl-6-O-(3,4,5-trimethoxybenzoyl)-*β*-D-glucopyranoside-1	[7]	
16-*β*-(*β*-D-Glucopyranosyl-2)-3,8,22-tri-hydroxy cholest-5,14,16,23-tetraene-1-*β*-yl-6-O-(3,4,5-trimethoxybenzoyl)-*β*-D-glucopyranoside-2		

## References

[1] Idu M., Omogbai E. K. I., Aghimien G. E., Amaechina F., Timothy O., Omonigho S. E. (2007). Preliminary phytochemistry, antimicrobial properties and acute toxicity of *Stachytarpheta jamaicensis* (L.) Vahl. leaves. *Trends in Medical Research*.

[2] Ramakrishnan K., Sivaranjani R. (2013). Pharmacognostical and phytochemical studies on stem of *Stachytarpheta jamaicensis* (L) Vahl. *International Research Journal of Pharmacy*.

[3] Joshi V., Sutar P., Karigar A., Patil S., Gopalakrishna B., Sureban R. (2010). Screening of ethanolic extract of *Stachytarpheta indica* L. (vahl) leaves for hepatoprotective activity. *International Journal of Research in Ayurveda and Pharmacy*.

[4] Meena R., Pitchai R. (2011). Evaluation of antimicrobial activity and preliminary phytochemical studies on whole plant of *Stachytarpheta jamaicensis* (L.) Vahl. *International Research Journal of Pharmacy*.

[5] Rahmatullah M., Jahan R., Azam F. M. S., Hossan S., Mollik M. A. H., Rahman T. (2011). Folk medicinal uses of verbenaceae family plants in Bangladesh. *African Journal of Traditional, Complementary and Alternative Medicines*.

[6] Vikrant A., Arya M. L. (2011). A review on anti-inflammatory plant barks. *International Journal of PharmTech Research*.

[7] Okwu D. E., Ohenhen O. N. (2010). Isolation and characterization of steroidal glycosides from the leaves of *Stachytarpheta Jamaicensis* Linn Vahl. *Der Chemica Sinica*.

[8] Sivaranjani R., Ramakrishnan K., Bhuvaneswari G. (2014). Pharmacognostic studies on root of *Stachytarpheta jamaicensis*. *International Letters of Natural Sciences*.

[9] Idu M., Erhabor J. O., Odia E. A. (2009). Morphological and anatomical studies of the leaf and stem of some medicinal plants:* Stachytarpheta jamaicensis* (L.) Vahl. And *S. cayennensis* (LC Rich) schau. *Ethnobotanical Leaflets*.

[10] Lakshmi P. V., Raju A. S. (2011). Psychophily in *Stachytarpheta jamaicensis* (L.) Vahl. (Verbenaceae). *Current Science*.

[11] Sivaranjani R., Ramakrishnan K., Bhuvaneswari G. (2013). Morpho-anatomical and preliminary phytochemical studies of the leaf of *Stachytarpheta jamaicensis* (L) Vahl. *International Journal of PharmTech Research*.

[12] Sulaiman M. R., Zakaria Z. A., Chiong H. S., Lai S. K., Israf D. A., Azam Shah T. M. (2009). Antinociceptive and anti-inflammatory effects of *Stachytarpheta jamaicensis* (L.) Vahl (Verbenaceae)in experimental animal models. *Medical Principles and Practice*.

[13] Jagadish N. R. N., Gopalkrishna B. (2008). Evaluation of analgesic activity of different extracts of *Stachytarpheta indica* L. (Vahl). *Biomed*.

[14] Valdés A. F.-C., Martínez J. M., Lizama R. S., Gaitén Y. G., Rodríguez D. A., Payrol J. A. (2010). In vitro antimalarial activity and cytotoxicity of some selected cuban medicinal plants. *Revista do Instituto de Medicina Tropical de São Paulo*.

[15] Putera I., Anis Shazura K. (2010). *Antimicrobial activity and cytotoxic effects of Stachytarpheta jamaicensis (L.) Vahl crude plant extracts [Master dissertation]*.

[16] Thomas R. P., Thomas M., Paul J., Mohan M. (2013). Antifungal activity of verbenaceae. *Biosciences Biotechnology Research Asia*.

[17] Suneetha P., Poornima S., Sumana K., Nidhi H., Puttaraju H. P. (2013). Comparative studies on antimicrobial and antifungal efficacy from *Bixa Orellana* L., *Lantana Camara* L., *Stachytarpheta jamaicensis* (L.)Vahl., *Hyptis Suaveolens* (L.) poit. With triclosan. *CIBTech Journal of Microbiology*.

[18] Ruma O. C., Zipagang T. B. (2015). Determination of secondary metabolites and antibacterial property of extract from the leaves of *Stachytarpheta jamaicensis* (L.) Vahl. *Journal of Medicinal Plants and Studies*.

[19] Brasileiro B. G., Pizziolo V. R., Raslan D. S., Jamal C. M., Silveira D. (2006). Antimicrobial and cytotoxic activities screening of some Brazilian medicinal plants used in Governador Valadares district. *Revista Brasileira de Ciências Farmacêuticas*.

[20] Halliwell B., Gutteridge J. M. C. (2007). *Free Radicals in Biology and Medicine*.

[21] Veskoukis A. S., Tsatsakis A. M., Kouretas D. (2012). Dietary oxidative stress and antioxidant defense with an emphasis on plant extract administration. *Cell Stress and Chaperones*.

[22] Álvarez E., Leiro J. M., Rodríguez M., Orallo F. (2004). Inhibitory effects of leaf extracts of *Stachytarpheta jamaicensis* (Verbenaceae) on the respiratory burst of rat macrophages. *Phytotherapy Research*.

[23] Sivaranjani R., Ramakrishnan K., Bhuvaneswari G. (2014). Evaluation of in vitro antioxidant activity and estimation of total phenol and flavonoids contents of various extracts of *Stachytarpheta jamaicensis*. *International Journal of Innovative Drug Discovery*.

[24] Dillard C. J., Bruce German J. (2000). Phytochemicals: nutraceuticals and human health. *Journal of the Science of Food and Agriculture*.

[25] Wan Rozianoor M. H., Nurol Eizzatie Y., Nurdiana S. (2014). Hypoglycemic and antioxidant activities of *Stachytarpheta jamaicensis* ethanolic leaves extract on alloxan-induced diabetic sprague dawley rats. *BioTechnology*.

[26] Charpignon C., Lesgourgues B., Pariente A. (2013). Peptic ulcer disease: one in five is related to neither *Helicobacter pylori* nor aspirin/NSAID intake. *Alimentary Pharmacology & Therapeutics*.

[27] Rodríguez L. A. G., Tacconelli S., Patrignani P. (2008). Role of dose potency in the prediction of risk of myocardial infarction associated with nonsteroidal anti-inflammatory drugs in the general population. *Journal of the American College of Cardiology*.

[28] Gutiérrez S. P., Sánchez M. A. Z., Gonzlález C. P., García L. A. (2007). Antidiarrhoeal activity of different plants used in traditional medicine. *African Journal of Biotechnology*.

[29] Meite S., N'Guessan J. D., Bahi C., Yapi H. F., Djaman A. J., Guede Guina F. (2009). Antidiarrheal activity of the ethyl acetate extract of *Morinda morindoides* in rats. *Tropical Journal of Pharmaceutical Research*.

[30] Sasidharan S., Latha L., Zuraini Z., Suryani S., Sangetha S., Shirley L. (2007). Antidiarrheal and antimicrobial activities of *Stachytarpheta jamaicensis* leaves. *Indian Journal of Pharmacology*.

[31] Ikewuchi J. C., Okaraonye C. C., Ogbonnaya E. A. (2009). Time course of the effect *Stachytarpheta jamaicensis* L. (Vahl.) on plasma sodium and potassium levels of normal rabbits. *Journal of Applied Sciences Research*.

[32] Idu M., Omogbai E. K. I., Amaechina F., Ataman J. E. (2006). Some cardiovascular effects of the aqueous extract of the leaves of *Stachytarpheta jamaicensis* L. Vahl. *International Journal of Pharmacology*.

[33] American Diabetes Association (2002). Evidence-based nutrition principles and recommendations for the treatment and prevention of diabetes and related complications. *Clinical Diabetes*.

[34] Martirosyan D. M., Miroshnichenko L. A., Kulakova S. N., Pogojeva A. V., Zoloedov V. I. (2007). Amaranth oil application for coronary heart disease and hypertension. *Lipids in Health and Disease*.

[35] Shen G. X. (2007). Lipid disorders in diabetes mellitus and current management. *Current Pharmaceutical Analysis*.

[36] Ikewuch C. J., Ikewuchi C. C. (2010). Alteration of plasma lipid profiles and atherogenic indices by *Stachytarpheta jamaicensis* L.. *Biokemistri*.

[37] Pandian C., Srinivasan A., Pelapolu I. C. (2013). Evaluation of wound healing activity of hydroalcoholic extract of leaves of *Stachytarpheta jamaicensis* in streptozotocin induced diabetic rats. *Der Pharmacia Lettre*.

[38] Nazar S., Ravikumar S., Williams G. P., Ali M. S., Suganthi P. (2009). Screening of Indian coastal plant extracts for larvicidal activity of *Culex quinquefasciatus*. *Indian Journal of Science and Technology*.

[39] Chariandy C. M., Seaforth C. E., Phelps R. H., Pollard G. V., Khambay B. P. S. (1999). Screening of medicinal plants from Trinidad and Tobago for antimicrobial and insecticidal properties. *Journal of Ethnopharmacology*.

[40] Robinson R. D., Williams L. A. D., Lindo J. F., Terry S. I., Mansingh A. (1990). Inactivation of strongyloides stercoralis filariform larvae in vitro by six Jamaican plant extracts and three commercial anthelmintics. *The West Indian Medical Journal*.

[41] Iroha I. R., Ilang D. C., Ayogu T. E., Oji A. E., Ugbo E. C. (2010). Screening for anti-typhoid activity of some medicinal plants used in traditional medicine in Ebonyi state, Nigeria. *African Journal of Pharmacy and Pharmacology*.

[42] Woradulayapinij W., Soonthornchareonnon N., Wiwat C. (2005). *In vitro* HIV type 1 reverse transcriptase inhibitory activities of Thai medicinal plants and *Canna indica* L. rhizomes. *Journal of Ethnopharmacology*.

[43] Caamal-Fuentes E., Torres-Tapia L. W., Simá-Polanco P., Peraza-Sánchez S. R., Moo-Puc R. (2011). Screening of plants used in Mayan traditional medicine to treat cancer-like symptoms. *Journal of Ethnopharmacology*.

[44] Rang H. P., Ritter J. M., Flower R. J., Henderson G. (2014). *Rang & Dale's Pharmacology: With Student Consult Online Access*.

[45] Idu M., Ataman J. E., Akhigbe A. O., Omogbai E. K. I., Amaechina F., Odia E. A. (2006). Effect of *Stachytarpheta jamaicensis* L. (Vahl.) on Wistar rats: serum biochemistry and ultrasonography. *Journal of Medical Sciences*.

[46] Ataman J. E., Idu M., Odia E. A. (2006). Histopathologic effects of *Stachytarpheta jamaicensis* (L.) Vahl on Wistar rats. *Pakistan Journal of Biological Sciences*.

[47] Ogie-Odia E. A., Ilechie M. N., Erhenhi A. H., Oluowo E. F. (2009). Effects of dose-related levels of powdered *Stachytarpheta jamaicensis* Vahl leaves on body weight and liver functions of albino rats. *Researcher*.

[48] Princely S., Basha N. S., Kirubakaran J. J., Dhanaraju M. D. (2013). Preliminary phytochemical screening and antimicrobial activity of aerial parts of *Stachytarpheta indica* L. (Vahl.). *Medicinal Plants*.

[49] Sahoo S. R., Dash R. R., Bhatnagar S. (2014). Phytochemical screening and bioevaluation of medicinal plant *Stachytarpheta indica* (L.) Vahl. *Pharmacology & Toxicology Research*.

[50] Jawad F. H., Doorenbosx N. J., Chengt P. C. (1977). Tarphetalin: a novel iridoid glucoside from *Stachytarpheta jamaicensis* (L.) Vahl. *Egyptian Journal of Pharmaceutical Science*.

[51] Chung K. S., Takeatsu K., Paul P. H. B., Ji-Xiang G. (1997). *International Collation of Traditional and Folk Medicine: Northeast Asia III*.

[52] Lin H. J., Chen A. H. (1976). Phytochemical study on *Stachytarpheta jamaicensis*. *Journal of the Chinese Agricultural Chemical Society*.

[53] Melita R. S., Castro O. (1996). Pharmacological and chemical evaluation of *Stachytarpheta jamaicensis* (Verbenaceae). *Revista de Biología Tropical*.

[54] Okwu D. E., Ohenhen O. N. (2009). Isolation, characterisation and antibacterial activity of lanostane triterpenoid from the leaves of *Stachyterpheta jamaicensis* Linn Vahl. *Der Pharma Chemica*.

